# Physiologic and mechanical responses to clustered vs. traditional sprint interval exercise approaches

**DOI:** 10.1007/s00421-025-05857-4

**Published:** 2025-06-29

**Authors:** Refik Çabuk, Yıldırım Kayacan, Juan Manuel Murias, Bettina Karsten

**Affiliations:** 1 Department of Coaching Education, Yaşar Doğu Faculty of Sport Sciences, Ondokuz Mayıs University, Samsun, Türkiye; 2 College of Health and Life Sciences, Hamad Bin Khalifa University, Doha, Qatar; 3 Faculty of Health, Pedagogy & Social Science, CBS University of Applied Science, Cologne, Germany; 4 Institute for Lifecourse Development, School of Human Sciences, Centre for Exercise Activity and Rehabilitation, University of Greenwich, London, United Kingdom

**Keywords:** Aerobic training, Aerobic capacity, Maximal oxygen consumption, Peak power output, Repeated sprint exercise

## Abstract

**Purpose:**

This study compared responses to a traditional 30-s all-out sprint interval exercise (SIE) session, compared to two SIE sessions divided into clusters, with the aim to assess which of these sessions would result in higher peak oxygen uptake (${\dot{\text{V}}}$O_2peak_), longer time at ${\dot{\text{V}}}$O_2_ ≥ respiratory compensation point (RCP), and greater peak power output during SIE (PPO_SIE_).

**Methods:**

Twelve trained males (19 ± 1 years; 176 ± 5 cm; 65.9 ± 6 kg; ${\dot{\text{V}}}$O_2max_: 54.0 ± 6.2 mL kg^−1^ min^−1^) performed three work-matched all-out cycling SIE sessions with a load of 7.5% body mass: (1) SIE30: 4 repetitions of 30-s work with 240-s recovery; (2) SIE15: 4 repetitions of 15-s work with 15-s recovery, plus 15-s work with 225-s recovery; (3) SIE10: 4 repetitions of 10-s work with 10-s recovery, plus 10-s work and 10-s recovery, plus 10-s work with 220-s recovery.

**Results:**

PPO_SIE_ for SIE30 (697 ± 71 W) was lower than for SIE15 (732 ± 63 W; *p* = 0.001) and SIE10 (752 ± 75 W; *p* = 0.001). ${\dot{\text{V}}}$O_2peak_ response for SIE30 (46.5 ± 6.6 mL kg^−1^ min^−1^) was lower than for SIE15 (51.9 ± 4.8 mL kg^−1^ min^−1^; *p* = 0.04) and SIE10 (50.9 ± 5.6 mL kg^−1^ min^−1^; *p* = 0.01). Time spent at ${\dot{\text{V}}}$O_2_ ≥ RCP was shorter for SIE30 (32.9 ± 35.9 s) compared to SIE15 (95.0 ± 52.0 s; *p* = 0.001) and SIE10 (62.9 ± 46.1 s; *p* = 0.010). No differences were identified for these variables between SIE15 and SIE10 (*p* = 0.270).

**Conclusion:**

Compared to the SIE30 session, the clustering-based SIE protocols resulted in higher PPO_SIE_ values, a greater ${\dot{\text{V}}}$O_2peak_ response, and longer time spent at ${\dot{\text{V}}}$O_2_ ≥ RCP. Thus, clustering methods can maximize the above-mentioned responses and be appealing alternatives to the traditional 30-s SIE session.

## Introduction

Sprint interval exercise (SIE) sessions involve short bursts (≤ 30-s) of maximal-intensity all-out exercise interspersed with passive or active recovery periods, commonly performed in a range of 4–6 bouts (for a review, see Gist et al. 2014). A widely used SIE protocol is the Wingate-based cycling (SIE30) (Burgomaster et al. 2008; Dal Pupo et al. 2021; Iannetta et al. 2022; for a review, see Vollaard et al. 2017), which typically comprises 4 min of recovery periods between bouts of 30-s all-out sprint efforts at a load equivalent to 5–10% of body mass (BM), depending on the characteristics of the population (Burgomaster et al. 2005; Inglis et al. 2024). The SIE30 protocol has been demonstrated as a time-efficient and effective protocol to increase aerobic fitness and obtain health benefits (Burgomaster et al. 2008; for a review, see Vollaard et al. 2017). However, the SIE30 protocol is physically taxing, as the all-out efforts produce the attainment of very high peak power output during SIE (PPO_SIE_) values within the first few seconds of the bout (especially when a pre-acceleration phase is included), with a steady decrease in power output (PO) during the remaining of the task as the task of keeping the high work rates becomes unsustainable (Balci et al. 2022; Demirarar and Cabuk 2025; Ozkaya et al. 2018; Pekünlü et al. 2016). A strategy to prevent the reduction in PPO_SIE_ during SIE is the use of varied types of repeated sprint exercise (RSE) with sprint durations typically ranging from 3-s to 10-s (Benítez-Flores et al. 2018; for a review, see Thurlow et al. 2023), and recovery durations varying from 15-s to 60-s (Benítez-Flores et al. 2018; Fiorenza et al. 2018; Shi et al. 2018) work to recovery ratios varying between 1:2.5 and 1:10 (Shi et al. 2018). RSE sessions based on SIE protocols have been utilized as a method to increase ${\dot{\text{V}}}$O_2max_ by promoting high PPO_SIE_ production (Ikutomo et al. 2018). The rapid decline in PO during SIE sessions can be partly attributed to the depletion of phosphocreatine (PCr) stores, as well as other factors contributing to the development of fatigue (for a review, see Allen et al. 2002; Duke and Steele 2001; for a review, see Westerblad et al. 2002). Specifically, PCr [which plays a critical role in the rapid resynthesis of adenosine triphosphate (ATP) during SIE sessions] quickly reduces due to its limited capacity. This leads to a shift toward greater reliance on the glycolytic and oxidative systems for ATP resynthesis within and between bouts as the exercise progresses (McCartney et al. 1986; Spriet et al. 1989). Therefore, during SIE30 sessions, there is a progressive decrease in PPO_SIE_, average PO, minimum PO, and cycling cadence (revolutions per minute; RPM) from the first to the final 30-s sprint work bout (Buchheit et al. 2012; Burgomaster et al. 2005; Chan and Burns 2013; Townsend et al. 2013; McCartney et al. 1986). This is important because maintaining a high PPO_SIE_ has been shown to be a more critical factor than total exercise volume in increasing maximal oxygen uptake (${\dot{\text{V}}}$O_2max_) following a sprint interval training (SIT) program (Hazell et al. 2010; Zelt et al. 2014).

Further, it is suggested that a high oxygen uptake (${\dot{\text{V}}}$O_2_) response and longer exercise time spent at high percentages of ${\dot{\text{V}}}$O_2max_ or at a ${\dot{\text{V}}}$O_2_ greater than that corresponding to the respiratory compensation point (${\dot{\text{V}}}$O_2RCP_) (i.e., within the severe intensity domain) are important acute stimuli for triggering aerobic adaptations (Faricier et al. 2025; Nakahara et al. 2020; Odden et al. 2024). Although evaluations of time spent in close proximity to ${\dot{\text{V}}}$O_2max_ are more common for high intensity interval training (Odden et al. 2024; Midgley et al. 2006; Norouzi et al. 2023; Wakefield and Glaister 2009), to the best of our knowledge, only one study evaluated this during SIE sessions (Shi et al. 2018). These researchers demonstrated that a high ${\dot{\text{V}}}$O_2_ is dependent on the duration of the recovery period within SIE sessions, and a reduction in recovery duration has been suggested as an important strategy for increasing the time spent at high %${\dot{\text{V}}}$O_2max_. Shi et al. (2018) in their study compared the traditional SIE30 protocol (8 × 30-s efforts with 240-s passive recovery) in a group of male endurance runners with an approach that matched the total work duration but consisted of 40 repetitions of all-out 6-s repeated sprints with different work to recovery ratios of 1:2.5 (15-s *r*), 1:5 (30-s *r*), and 1:10 (30-s *r*) (Shi et al. 2018). The results indicated that shorter recovery durations led to more time spent above 80% of ${\dot{\text{V}}}$O_2max_. However, as the recovery duration shortened, greater decreases in PPO_SIE_ and average PO were observed (Shi et al. 2018). La Monica et al. (2016) evaluated a group of recreationally trained males using fixed 6-s work durations and varying recovery times with work to recovery ratios of 1:2 (12-s active recovery), 1:3 (18-s active recovery), and 1:4 (24-s active recovery). They showed greater ${\dot{\text{V}}}$O_2_ responses as recovery durations shortened. However, this led to lower total work, PPO_SIE_, and average PO values. Finally, Benítez-Flores et al. (2018) demonstrated that in physically active young men, an RSE protocol consisting of 6 × 5-s all-out repeated sprints with 24-s active recovery elicited higher values for heart rate (HR), ${\dot{\text{V}}}$O_2_, average PO, minimum PO, and total work compared to an equivalent-volume SIE protocol consisting of 4 × 20-s all-out sprints with 120-s active recovery periods, while yielding similar PPO_SIE_ responses. Understanding how the combination of work and recovery affect physiologic responses is crucial for optimizing SIE protocols tailored to specific training goals.

Although there are infinite number of work to recovery combinations that could alter acute responses and chronic adaptations to sprint interval exercise (especially when considering RSE models as part of SIE sessions), the SIE30 is arguably the most widely studied model in research (Burgomaster et al. 2008; Dal Pupo et al. 2021; Iannetta et al. 2022; for a review, see Vollaard et al. 2017). Further, some studies have evaluated the physiologic responses to SIE sessions of different work time durations (Hazell et al. 2010; La Monica et al. 2016; Lloria-Varella et al. 2023) with similar (Lloria-Varella et al. 2023) or varied (Benítez-Flores et al. 2018; Vardarli et al. 2021; Shi et al. 2018) work to recovery ratios. However, no study has evaluated how a clustering approach to SIE sessions that modulates the work and recovery durations can affect all, PPO_SIE_ and ${\dot{\text{V}}}$O_2peak_ responses and the time spent at high % of ${\dot{\text{V}}}$O_2max_ or ≥ ${\dot{\text{V}}}$O_2RCP_. Given that SIE sessions are performed within the extreme exercise intensity domain (Ozkaya et al. 2024), the recovery between bouts predominantly depends on the rate of PCr resynthesis (Bogdanis et al. 1995 and for a review, see 1996), which following a maximal sprint can be replenished in half after ~ 20–30-s (Bogdanis et al. 1995). Thus, dividing a 30-s work bout into 2 or 3 work bout clusters to provide recovery durations within sprints that allow for partial recovery of PCr while still maintaining a high cardiovascular strain might allow for greater PO values and % of ${\dot{\text{V}}}$O_2max_ or ≥ ${\dot{\text{V}}}$O_2RCP_ responses to be observed. Such approach should result in greater PPO_SIE_ and maintenance of PO values within and between bouts. Furthermore, a relatively shorter recovery duration between bouts to maintain the work to recovery ratio may result in a greater ${\dot{\text{V}}}$O_2_ response and a longer time spent at high % of ${\dot{\text{V}}}$O_2max_ or ≥ ${\dot{\text{V}}}$O_2RCP_.

Thus, the present study compared the traditionally performed SIE30 protocol with two experimental protocols of equal workload by splitting work bouts into either 2 × 15-s (SIE15) or 3 × 10-s (SIE10) clusters and manipulating intra- and inter work bout recovery durations. We hypothesized that, compared to SIE30, both experimental protocols, SIE15 and SIE10, would result in: (i) greater PPO_SIE_ values, (ii) greater ${\dot{\text{V}}}$O_2_ responses, and (iii) longer time spent at high % of ${\dot{\text{V}}}$O_2max_ or ≥ ${\dot{\text{V}}}$O_2RCP_ than those obtained in the traditional SIE30 protocol.

## Materials and methods

### Participants and ethical approval

Twelve recreationally trained male athletes [age: 20 ± 1 years; stature: 176 ± 5 cm; BM: 66 ± 6 kg; ${\dot{\text{V}}}$O_2max_: 54.0 ± 6.2 mL kg^−1^ min^−1^; Peak power output from maximal graded exercise test (PPO_GXT_): 294 ± 16 W; maximal heart rate from maximal graded exercise test (HR_max_): 195 ± 3 beat min^−1^] participated in the study. Participants had an average of 7 ± 2 years of training experience, and their weekly training frequency was 5.3 ± 1.4 sessions. All participants performed at least 2–4 endurance training sessions per week in their programs. The study was approved by the Clinical Research Ethics Committee of Ondokuz Mayıs University in accordance with the Declaration of Helsinki. Participants were fully informed about the nature and risks of the study prior to providing written informed consent. They were asked not to consume any beverages containing more than 60 mg of caffeine during the study. Participants were requested to maintain their regular diet during the study, to refrain from eating at least 3 h before any testing and exercise session and to avoid any strenuous activity at least 24 h prior. None of the participants had any injuries or were taking any medication, and they had no known systemic diseases (e.g., cardiovascular, pulmonary, metabolic, muscular, or coronary).

### Experimental protocol

**Fig. 1:**
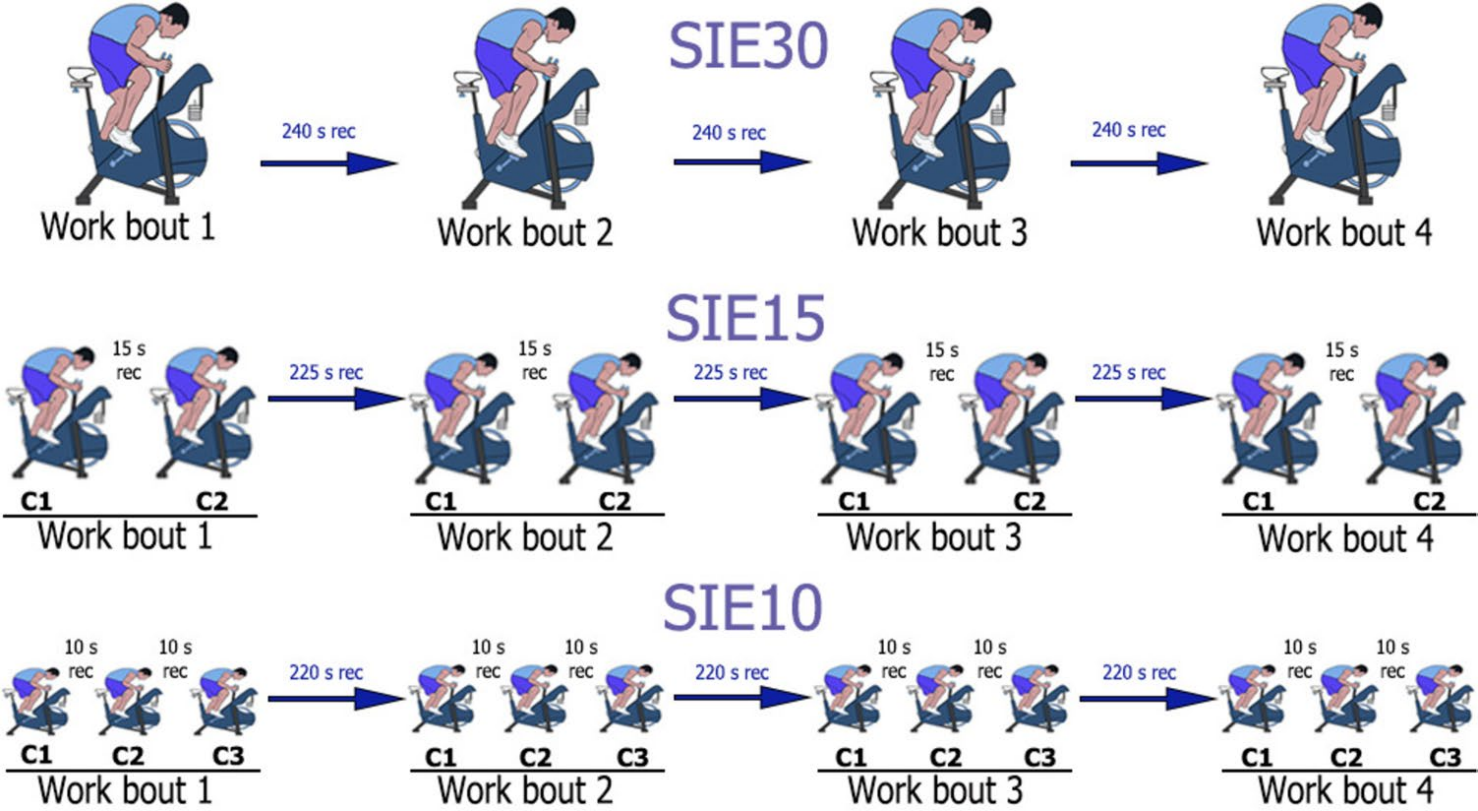
Presentation of standardized and experimental SIE protocols. *C1* 1st cluster, *C2* 2nd cluster, *C3* 3rd cluster, *rec* recovery; SIE30 protocol: 4 × 30-s work bout + 240-s active recovery; SIE15 protocol: 4 × 15-s work bout + 15-s active recovery + 15-s work bout + 225-s active recovery; SIE10 protocol: 4 × 10-s work bout + 10-s active recovery + 10-s work bout + 10-s active recovery + 10-s work bout + 220-s active recovery

This study required a total of five visits to the laboratory. To prevent the potential effects of circadian rhythm (Souissi et al. 2004), each participant was consistently tested at approximately the same time of day (± 1 h). All visits were separated by a minimum of 72 h. Laboratory conditions were stable in a range of 22–24 °C and 55–60% humidity. Both graded exercise test (GXT) and SIE protocols were performed on the same ergometer (Peak Bike 894, Monark, Vansbro, Sweden). During the first visit, stature and BM measurements were taken (Seca 767, Germany), and participants were familiarized with the procedures as described below to minimize learning effects (see below for details). The saddle and handlebar were adjusted to suit each participant, and settings were replicated during subsequent visits (Priego Quesada et al. 2017). During visit two, participants performed a GXT to determine ${\dot{\text{V}}}$O_2max_, PPO_GXT_ and maximal heart rate (HR_max_). During visits three to five, participants completed the three SIE protocols in a randomized order as described below. The SIE30 protocol consisted of four work bouts, each lasting 30 s with 240-s recovery periods (see below for details on work to recovery ratio for each SIE session). This 30-s work bout was used as the basis for developing two other SIE protocols using a cluster-based approach: SIE15 and SIE10. In the SIE15 protocol, the 30-s work bout was divided into two clusters of 15 s each, whereas in the SIE10 protocol, it was divided into three clusters of 10 s each. In this arrangement, the two clusters in the SIE15 protocol and the three clusters in the SIE10 protocol represent one work bout. Consequently, all protocols were evaluated based on a total of four work bouts. In addition, the ${\dot{\text{V}}}$O_2peak_ values corresponding to the eight work bouts and active recovery periods in the SIE15 protocol, as well as the 12 work bouts and the recovery periods in the SIE10 protocol, were also analyzed. Figure 1 provides a detailed visualization of the differences in configuration between the protocols.

#### Standardized SIE warm-up protocol

For the warm-up protocol, participants pedaled at a cadence of 70–80 RPM against a load of 1 kg for 5 min. During the last 5-s of the 3rd, 4th, and 5th minutes, participants had to pedal at their highest possible cadence against a load of 2 kg.

#### Familiarization session

The familiarization session was divided into two parts. In part 1, participants performed eight work bouts of 5-s all-out efforts against a standardized resistance of 7.5% BM with 40-s active recovery periods of unloaded cycling at 50 RPM between work bouts. Five minutes after the completion of part 1, in part 2, participants performed a 30-s all-out effort against the standardized resistance described above.

### Procedures and data analysis

#### Maximal graded exercise test

The GXT included a baseline period of 2 min at 85 W. Thereafter, the load was progressively increased by 0.3 kg every minute (equivalent to 25 W min^−1^) until volitional exhaustion. Participants were instructed to maintain a constant cadence of ~ 85 RPM throughout the test. The test was terminated when cadence fell below 80 RPM for more than 10-s despite strong verbal encouragement. At least two of the following criteria were used to verify a maximal test: (i) HR ≥ 90% of age-predicted HR maximum, (ii) a VO_2_ increase of < 150 mL min^−1^ despite increasing PO, (iii) a respiratory exchange ratio (RER) value of 1.15 or higher and iv) an rating of perceived exertion (RPE) level of 19 or 20 (Ferretti 2014; for a review, see Howley et al. 1995). The highest moving 10-s averages of ${\dot{\text{V}}}$O_2_ and HR were considered as ${\dot{\text{V}}}$O_2max_ and HR_max_, respectively. To determine the PPO_GXT_ more accurately during the GXT, the time at the final workload stage was taken into account. The PPO_GXT_ was calculated as follows:

$$\text{PO at the last completed stage}+\left(\frac{\text{Seconds sustained at the uncompleted stage}}{60\text{ s}}\times 25\, \text{W}\right)$$

(Adami et al. 2013).

### Respiratory compensation point

${\dot{\text{V}}}$O_2RCP_ was determined by two independent and experienced evaluators. Minute ventilation (${\dot{\text{V}}}$_E_), carbon dioxide production (${\dot{\text{V}}}$CO₂), RER, end-tidal O_2_ pressure (P_ET_O₂), and end-tidal carbon dioxide pressure (P_ET_CO_2_), ${\dot{\text{V}}}$_E_/${\dot{\text{V}}}$O_2_ and ${\dot{\text{V}}}$_E_/${\dot{\text{V}}}$CO_2_ were plotted against ${\dot{\text{V}}}$O_2_ during the GXT for the estimation of ${\dot{\text{V}}}$O_2RCP_ (Keir et al. 2022). ${\dot{\text{V}}}$O_2RCP_ was defined as the point at which P_ET_CO_2_ abruptly declined following a period of isocapnic buffering. This point was confirmed by the simultaneous presence of a second breakpoint in the relationship of ${\dot{\text{V}}}$_E_, ${\dot{\text{V}}}$_E_/${\dot{\text{V}}}$O₂, and ${\dot{\text{V}}}$_E_/${\dot{\text{V}}}$CO₂ against ${\dot{\text{V}}}$O₂, as well as a first breakpoint in the relationship of ${\dot{\text{V}}}$_E_ against ${\dot{\text{V}}}$CO_2_.

#### Sprint interval protocols

The total duration of work bouts (120 s) and recovery periods (720 s) were equalized between the SIE30 and the experimental SIE protocols (Fig. 1). The load was standardized to 7.5% of BM. Following the standardized warm-up and a 5-min resting period, participants performed one of the three SIE protocols in a randomized order. The SIE30 protocol consisted of four sets of 30-s all-out work bouts followed with 240-s active recovery periods (4 × 30-s work + 240-s recovery). SIE15 consisted of four sets, each including two 15-s all-out work bouts separated by a 15-s intra-interval active recovery (4 × 15-s work + 15-s recovery + 15-s work), followed by 225-s of active recovery. This sequence (15-s work + 15-s recovery + 15-s work + 225-s recovery) resulted in a total of eight 15-s all-out bouts. SIE10 involved four sets, each including three 10-s all-out work bouts separated by 10-s intra-interval active recoveries (4 × 10-s work + 10-s recovery + 10-s work + 10-s recovery + 10-s work), followed by 220-s of active recovery. This sequence resulted in a total of 12 10-s all-out bouts. All SIE protocols maintained a 1:8 work to recovery ratio (i.e., 30 s of work with 240-s of the recovery) for equal workload (Fig. 1).

SIE protocols were initiated at a cadence of 120 RPM. Upon reaching 120 RPM, the load of the ergometer basket automatically dropped onto the flywheel, time started, and participants were required to maintain the fastest possible cadence. Following the completion of each work bout, the active recovery period automatically started. The resistance was lifted off within a maximally 2 to 3-s to stop the flywheel from spinning due to inertia. Participants performed unloaded cycling for the respective the recovery period at 50 RPM before the start of a subsequent work bout. Participants were verbally encouraged to perform a maximum effort for each work bout. Participants were instructed to maintain a seated position throughout all SIE protocols to avoid potential effects from postural changes.

#### Mechanical power outputs and total work calculations

Four different mechanical indices were calculated for each bout within individual SIE protocols. These are: (i) PPO_SIE_, defined as the highest power output achieved during the work bout period; (ii) average PO, considered as the average of power output obtained during the work bout; (iii) minimum PO, defined as the lowest mechanical power achieved during the work bout period; (iv) Fatigue Index (FI), calculated as: FI = [(PPO_SIE_-Minimum PO)/PPO_SIE_] × 100. In addition, the total amount of work performed (Average PO × work bout duration) was calculated in kilojoules (kJ) for each SIE protocol separately.

#### Physiologic measurements

Expired gasses were collected continuously breath-by-breath using a PNOE analyser (ENDO Medical, Palo Alto, CA). The ergometer was calibrated before each test according to the manufacturer’s instructions. The data collected were automatically converted into 5-s averages to facilitate analysis. This averaging was performed by the device’s software, which is equipped with automatic smoothing techniques.

HR was continuously monitored using an HR monitor (T31, Polar, Kempele, Finland) paired with the gas analyser. Prior to each test, the gas analyser was calibrated according to the manufacturer’s guidelines. Respiratory gas and HR responses were recorded continuously during SIE protocols. Blood lactate for the SIE protocols was measured using 25-µL (microliter) blood samples collected from the fingertips by applying heparinised hematocrit capillary tubes. Samples were analyzed using a YSI 1500 Sport Lactate Analyser (YSI Inc., Yellow Springs, USA). Blood lactate was collected at baseline before the warm-up and minutes 1, 3, and 5 after completion of each SIE protocol. The highest blood lactate value obtained after completion of any of the SIE protocols was accepted as the maximum blood lactate concentration ([La_max_]). The difference between [La_max_] and resting blood lactate concentration was recorded as delta blood lactate ([ΔLa]). In addition, a 15-point Borg scale (i.e., 6–20) was used to measure RPE within each SIE protocol, with the RPE score being recorded within the first 10 s immediately after the completion of the final work bout of the protocol.

#### Physiologic and time calculations at high %${\dot{\text{V}}}$O_2max_ analysis

The highest values of ${\dot{\text{V}}}$O_2_ and HR responses for each of the SIE protocols were calculated based on the 10-s moving highest average recorded at any time during SIE (either during work bouts or active recovery periods as the duration of the work was shorter than even the fastest expected ${\dot{\text{V}}}$O_2_ kinetics response (Murias et al. 2011; Grey et al. 2015) and referred to as ${\dot{\text{V}}}$O_2peak_ and HR_peak_ respectively.

For each SIE bout and recovery period, the highest ${\dot{\text{V}}}$O_2_ responses were similarly calculated. The highest ${\dot{\text{V}}}$O_2_ value obtained from the four, eight, or 12 work bouts and recovery periods performed in the SIE30, SIE15, and SIE10 protocols, respectively, was considered as the ${\dot{\text{V}}}$O_2peak_ for that condition and are presented in Table 2.

In the SIE15 and SIE10 protocols, two and three clusters of short work bouts, respectively, were considered equivalent to a single 30-s work bout. Consequently, both experimental protocols were evaluated based on a total of four 30-s work bouts. The ${\dot{\text{V}}}$O_2average_ during the work period was determined based on the entire 30-s work bout, while the ${\dot{\text{V}}}$O_2average_ during the recovery period was calculated based on the entire 4 min period. These ${\dot{\text{V}}}$O_2peak_ values, along with ${\dot{\text{V}}}$O_2average_ values, are presented in Table 3.

Research has demonstrated that spending time at ≥ 80% ${\dot{\text{V}}}$O_2max_ is effective for inducing meaningful aerobic performance and health adaptations (Burgomaster et al. 2005; Freese et al. 2013). Therefore, and despite the established limitations of this approach (Iannetta et al. 2020), this intensity was used to examine time spent at high percentages of ${\dot{\text{V}}}$O_2max_. The total time spent at ≥ 80% ${\dot{\text{V}}}$O_2max_ within the SIE protocols was consequently calculated by summing the 5-s intervals at this intensity (Freese et al. 2013; Shi et al. 2018).

### Statistical analysis

An a priori analysis revealed that a minimum sample size of 12 participants was required at a statistical power equal to 0.80 (two-tailed *α* = 0.05) and an effect size equal to 0.8. Data were first examined for normal distribution using the Shapiro–Wilk test. Group differences for the variables of interest were analyzed using a repeated measures ANOVA. When the differences were detected, Bonferroni post-hoc analysis was used to interpret where the differences existed. Partial eta squared ($\eta_{{\text{p}}}^{2}$) values were calculated for ANOVA comparisons, with effect sizes classified as small (< 0.02), medium (0.02–0.26), or large (> 0.26). A paired t-test was conducted to compare ${\dot{\text{V}}}$O_2_ values between the work bout and recovery phases within the SIE protocols. In addition, effect sizes (ES) of the differences in pairwise comparisons were calculated according to Cohen’s d coefficient (Cohen 1998). Cohen’s *d* was categorized as trivial effect (< 0.2), small effect (0.2–0.5), medium effect (0.5–0.8) and large effect (> 0.8). Statistical difference was accepted at *p* < 0.05. Results are reported as mean ± SD.

## Results

### Peak oxygen uptake between the SIE protocols and maximal oxygen consumption obtained from GXT

There were differences in the highest ${\dot{\text{V}}}$O_2_ responses between the SIE protocols and ${\dot{\text{V}}}$O_2max_ obtained from GXT (*F*(3, 33) = 5.0, *p* = 0.006, $\eta_{{\text{p}}}^{2}$ = 0.31). The highest ${\dot{\text{V}}}$O_2_ responses during the SIE corresponded to 86.1% ${\dot{\text{V}}}$O_2max_ (*p* = 0.024, ES = 1.04), 96.1% ${\dot{\text{V}}}$O_2max_ (*p* = 1.0, ES = 0.24), and 94.2% ${\dot{\text{V}}}$O_2max_ (*p* = 0.591, ES = 0.52) values for the SIE30, SIE15 and SIE10 protocols, respectively (Table 1).

**Table 1:** Physiologic and time responses to the sprint interval protocols

Variable	SIE30	SIE15	SIE10
${\dot{\text{V}}}$O_2peak_ (mL kg^−1^ min^−1^)	46.5 ± 6.5	51.9 ± 4.8^a^	50.9 ± 5.6^a^
${\dot{\text{V}}}$O_2peak_ at work bout (mL kg^−1^ min^−1^)	46.1 ± 6.5	49.75 ± 5.6	48.4 ± 6.6
${\dot{\text{V}}}$O_2peak_ at recovery (mL kg^−1^ min^−1^)	42.5 ± 6.6	50.0 ± 5.0^a^	48.5 ± 8.1^a^
Time spent at ≥ RCP (s)	32.9 ± 35.9	95.0 ± 52.0^a^	76.6 ± 48.9^a^
Time spent at ≥ 80% ${\dot{\text{V}}}$O_2max_ (s)	27.9 ± 28.4	76.6 ± 36.8^a^	62.9 ± 46.1^a^
Relative time spent at ≥ RCP (%)	27.4 ± 25.6	79.1 ± 37.3^a^	63.9 ± 45.5^a^
Relative time spent at ≥ 80% ${\dot{\text{V}}}$O_2max_ (%)	23.3 ± 23.7	63.9 ± 30.7^a^	52.4 ± 38.5^a^
HR_peak_ (beat min^−1^)	180 ± 8	182 ± 10	181 ± 9
Baseline lactate (mmol L^−1^)	1.4 ± 0.2	1.4 ± 0.3	1.41 ± 0.2
ΔLa (mmol L^−1^)	15.0 ± 3.2	15.2 ± 2.5	15.7 ± 3.4
Maximal lactate (mmol L^−1^)	16.4 ± 3.1	16.6 ± 2.4	17.1 ± 3.3
RPE	19 ± 1	19 ± 1	19 ± 1

^a^Different from the SIE30 protocol

### Peak oxygen uptake during bout periods among the SIE protocols

There were no differences in bout ${\dot{\text{V}}}$O_2peak_ responses among the SIE protocols (*F*(2, 10) = 2.23, *p* = 0.158, $\eta_{{\text{p}}}^{2}$ = 0.31). Bout ${\dot{\text{V}}}$O_2peak_ responses corresponded to 85.3%, 92.1%, and 89.6% of ${\dot{\text{V}}}$O_2max_ for the SIE30, SIE15, and SIE10 protocols, respectively (*p* values ranging from 0.520 to 0.582, ES values ranging from 0.16 to 0.63) (Table 1).

### Peak oxygen uptake during the recovery periods among the SIE protocols

There were differences in the recovery ${\dot{\text{V}}}$O_2peak_ responses among the SIE protocols (*F*(2, 10) = 9.83, *p* = 0.004, $\eta_{{\text{p}}}^{2}$ = 0.66). The highest recovery ${\dot{\text{V}}}$O_2peak_ responses were observed in the SIE15 and SIE10 protocols, corresponding to 92.5% and 90% of ${\dot{\text{V}}}$O_2max_, respectively (*p* = 0.563, ES = 0.17 for SIE15 vs. SIE10). In contrast, the lowest recovery ${\dot{\text{V}}}$O_2peak_ response was obtained in the SIE30 protocol, equating to 78.7 of ${\dot{\text{V}}}$O_2max_ (p = 0.003, ES = 1.07 for SIE15 vs. SIE30 and *p* = 0.008, ES = 0.93 for SIE10 vs. SIE30) (Table 1).

### Time spent at ≥ 80% of ${\dot{\text{V}}}$O_2max_

There were differences in the time spent at ≥ 80% of ${\dot{\text{V}}}$O_2max_ across the SIE protocols (*F*(2, 10) = 6.74, *p* = 0.014, $\eta_{{\text{p}}}^{2}$ = 0.57) The percent of time spent at ≥ 80% of ${\dot{\text{V}}}$O_2max_ was lower for SIE30, compared to SIE15 (*p* = 0.008, ES = 0.93) and SIE10 (*p* = 0.013, ES = 0.86), with no differences observed between SIE15 and SIE10 (*p* = 0.408, ES = 0.25) (Table 1).

### Time spent at ≥ ${\dot{\text{V}}}$O_2_ level corresponding to the respiratory compensation point

There were differences in the time spent at ≥ ${\dot{\text{V}}}$O_2RCP_ across the SIE protocols (*F*(2, 22) = 9.80, *p* = 0.001, $\eta_{{\text{p}}}^{2}$ = 0.47). The percent of time spent at ≥ ${\dot{\text{V}}}$O_2RCP_ was lower for SIE30, compared to SIE15 (*p* = 0.001, ES = 1.23) and SIE10 (*p* = 0.010, ES = 0.98), with no differences observed between SIE15 and SIE10 (*p* = 0.270, ES = 0.33) (Table 1).

### Comparison of the ${\dot{\text{V}}}$O_2peak_ responses obtained from the work bout and the recovery periods of clusters in each set in SIE15 and SIE10 protocols

In the SIE15 protocol, the ${\dot{\text{V}}}$O_2peak_ responses obtained during the active recovery periods of the 1st work bout in the 1st, 2nd, and 3rd sets were higher than the ${\dot{\text{V}}}$O_2peak_ responses obtained during the corresponding work bout periods (*p* = 0.001–0.035, ES = 0.70–1.43). In addition, the ${\dot{\text{V}}}$O_2peak_ responses obtained during the work bout periods of the 2nd work bout in the 1st, 2nd, 3rd, and 4th sets were higher than the ${\dot{\text{V}}}$O_2peak_ responses obtained during the corresponding recovery period (*p* = 0.003–0.015, ES = 0.83–1.16).

In the SIE10 protocol, the only cluster where the ${\dot{\text{V}}}$O_2peak_ response during the work bout period was higher than that during the recovery period was in the 2nd cluster of the 1st set (*p* = 0.021, ES = 0.78). Conversely, the only cluster where the ${\dot{\text{V}}}$O_2peak_ response during the recovery period was higher than that during the work bout period was in the 1st cluster of the 2nd set (*p* = 0.041, ES = 0.67). In the remaining clusters of the other sets, the ${\dot{\text{V}}}$O_2peak_ responses during the work bout and the recovery periods were similar (*p* = 0.051–0.94; ES = 0.02–0.63) (Table 2).

**Table 2:** Comparison of the ${\dot{\text{V}}}$O_2peak_ response (mL kg^−1^ min^−1^) obtained from work bout and active recovery periods of clusters in each set in the SIE30, SIE15 and SIE10 protocols

Variables		SIE30	SIE15		SIE10		
Set	Period	Cluster 1	Cluster 1	Cluster 2	Cluster 1	Cluster 2	Cluster 3
1	Work bout	39.6 ± 6.6	32.0 ± 6.3^b^	43.5 ± 5.4^b^	36.0 ± 6.8	42.5 ± 5.5^c^	37.6 ± 9.1
	Recovery	37.3 ± 5.4	43.2 ± 7.9^b^	39.7 ± 6.6^b^	36.2 ± 8.2	38.4 ± 7.4^c^	35.7 ± 7.2
2	Work bout	45.3 ± 9.6^a^	39.8 ± 5.2^b^	45.8 ± 6.7^b^	34.7 ± 7.4^c^	43.7 ± 7.2	42.4 ± 6.2
	Recovery	38.3 ± 8.2^a^	45.1 ± 4.6^b^	40.7 ± 6.5^b^	42.8 ± 8.9^c^	40.9 ± 6.2	40.7 ± 6.7
3	Work bout	44.3 ± 7.1^a^	39.8 ± 5.8^b^	46.4 ± 7.2^b^	36.3 ± 4.5	42.8 ± 7.6	39.0 ± 6.9
	Recovery	37.0 ± 6.9^a^	47.0 ± 6.4^b^	40.3 ± 6.9^b^	42.7 ± 9.9	42.4 ± 5.4	39.4 ± 7.4
4	Work bout	42.2 ± 8.2^a^	41.1 ± 6.7	44.4 ± 7.8^b^	38.3 ± 5.1	42.6 ± 7.6	37.6 ± 4.9
	Recovery	34.7 ± 6.5^a^	44.3 ± 8.2	39.7 ± 7.4^b^	38.6 ± 6.1	42.3 ± 8.1	36.6 ± 6.8

^a^The highest ${\dot{\text{V}}}$O_2_ responses obtained from the work bout and active recovery period of each set in the SIE30 protocol

^b^The highest ${\dot{\text{V}}}$O_2_ responses obtained from the work bout and active recovery period of each set in the SIE15 protocol

^c^The highest ${\dot{\text{V}}}$O_2_ responses obtained from the work bout and active recovery period of each set in the SIE10 protocol

### Comparison of the ${\dot{\text{V}}}$O_2peak_ responses from the work bout and the recovery periods of each set of the SIE protocols

Within the SIE30 protocol, the ${\dot{\text{V}}}$O_2peak_ responses during the 2nd, 3rd, and 4th work bouts were higher than the ${\dot{\text{V}}}$O_2peak_ responses during the corresponding 2nd, 3rd, and 4th recovery periods (*p* = 0.001–0.009, ES = 0.91–1.36). In the SIE10 protocol, only the ${\dot{\text{V}}}$O_2peak_ response during the 1st work bout was higher than the ${\dot{\text{V}}}$O_2peak_ response during its corresponding recovery period (*p* = 0.009, ES = 0.90) (Table 3).

**Table 3:** ${\dot{\text{V}}}$O_2peak_ (mL kg^−1^ min^−1^) and ${\dot{\text{V}}}$O_2average_ (mL kg^−1^ min^−1^) responses from the work bout and active recovery periods of each set of the SIE protocols

Set	SIE30		SIE15		SIE10	
	Work bout	Recovery	Work bout	Recovery	Work bout	Recovery
${\dot{\text{V}}}$O_2peak_						
1	39.6 ± 6.6	37.3 ± 5.4	44.3 ± 4.6	45.5 ± 5.7^a^	43.8 ± 5.9	40.4 ± 5.7^e^
2	45.3 ± 9.6	38.3 ± 8.2^e^	46.4 ± 6.8	46.1 ± 3.2^b^	45.4 ± 6.9	44.6 ± 7.6
3	44.3 ± 7.1	37.0 ± 6.9^e^	47.3 ± 6.2	47.2 ± 6.5^c^	43.4 ± 7.6	45.8 ± 8.2^c^
4	42.2 ± 8.2	34.7 ± 6.5^e^	47.7 ± 6.6	45.6 ± 6.9^d^	43.8 ± 6.7	43.5 ± 7.0^d^
${\dot{\text{V}}}$O_2average_						
1	33.0 ± 5.3	18.1 ± 2.9^f^	33.2 ± 3.1	20.5 ± 2.8^f^	35.9 ± 5.4	18.9 ± 3.3
2	37.7 ± 7.5	18.0 ± 2.9	38.9 ± 4.3	21.2 ± 2.3^g^	37.6 ± 5.9	20.5 ± 3.2^g^
3	38.8 ± 7.1	17.8 ± 2.1	39.1 ± 4.9	21.7 ± 2.8^h^	37.5 ± 6.7	20.7 ± 3.6^h^
4	37.0 ± 7.3	15.7 ± 3.2^ı^	39.3 ± 6.2	19.0 ± 2.5^ı^	36.8 ± 6.3	17.2 ± 2.8^ı^

^a^SIE30 protocol was lower than other protocols in ${\dot{\text{V}}}$O_2peak_ response of the first active recovery period of SIE protocols

^b^SIE30 protocol was lower than other protocols in ${\dot{\text{V}}}$O_2peak_ response of the second active recovery period of SIE protocols

^c^SIE30 protocol was lower than other protocols in ${\dot{\text{V}}}$O_2peak_ response of the third active recovery period of SIE protocols

^d^SIE30 protocol was lower than other protocols in ${\dot{\text{V}}}$O_2peak_ response of the fourth active recovery period of SIE protocols

^e^ ${\dot{\text{V}}}$O_2peak_ during work bout is higher than ${\dot{\text{V}}}$O_2peak_ during active recovery

^f^The ${\dot{\text{V}}}$O_2average_ response of the first active recovery period of SIE protocols was different only between SIE30 and SIE15

^g^SIE30 protocol was different from other protocols in ${\dot{\text{V}}}$O_2average_ response of the second active recovery period of SIE protocols

^h^SIE30 protocol was different from other protocols in ${\dot{\text{V}}}$O_2average_ response of the third active recovery period of SIE protocols

^I^Three SIE protocols are different from each other in ${\dot{\text{V}}}$O_2average_ response of the third active recovery period of SIE protocols

### Peak heart rate

No differences were observed in HR_peak_ among the SIE protocols (*F*(2, 22) = 1.96, *p* = 0.164, $\eta_{{\text{p}}}^{2}$ = 0.151) (Table 1).

### Maximal and delta lactate responses

No differences were observed in [La_max_] (*F*(2, 22) = 0.489, *p* = 0.620, $\eta_{{\text{p}}}^{2}$ = 0.043) and ΔLa (*F*(2, 22) = 0.475, *p* = 0.628, $\eta_{{\text{p}}}^{2}$ = 0.041) among the SIE protocols (Table 1).

#### RPE

There were no differences in RPE (*F*(2, 22) = 1.25, *p* = 0.306, $\eta_{{\text{p}}}^{2}$ = 0.102) values among the SIE protocols (Table 1).

### Mechanical responses

#### Peak power output

There were differences in the PPO_SIE_ across the SIE protocols (*F*(2, 22) = 14.5, *p* = 0.001, $\eta_{{\text{p}}}^{2}$ = 0.57). PPO_SIE_ obtained from the SIE30 protocol was 5.0% lower compared to the SIE15 (*p* = 0.002, ES = 1.38), and 7.5% lower compared to the SIE10 protocol (*p* = 0.003, ES = 1.29) (Table 4). The differences in PPO_SIE_ were not different for SIE10 compared to SIE15 (*p* = 0.252, ES = 0.55) (Table 4).

**Table 4:** Mechanical responses expressed as the average of four sets of SIE30, SIE15 and, SIE10, taking clustering into account for each SIE session

Variables	SIE30	SIE15	SIE10
PPO_SIE_ (W)	697 ± 71	732 ± 63^a^	752 ± 75^a^
Average PO (W)	507 ± 36	518 ± 43^b^	532 ± 44.7^ab^
Minimum PO (W)	305 ± 45.7	314 ± 43.9^b^	335 ± 54.7^ab^
Fatigue Index (%)	55.0 ± 9.8	56.9 ± 5.6	55.2 ± 6.7
Total work done (kJ)	60.8 ± 4.3	62.2 ± 5.2^a^	63.8 ± 5.3^ab^

^a^Different from the SIE30 protocol

^b^Difference between the SIE15 and SIE10 protocols

#### Average power output

Differences in average PO were observed among the SIE protocols (*F*(2, 22) = 11.2, *p* = 0.001, $\eta_{{\text{p}}}^{2}$ = 0.51). average PO in the SIE10 protocol was 4.7% higher than in SIE30 (*p* = 0.006, ES = 1.15) and 2.6% higher than in SIE15 (*p* = 0.005, ES = 1.20). However, there was no difference between the SIE30 and SIE15 protocols (*p* = 0.235, ES = 0.56) (Table 4).

#### Total work

Differences in total work were observed among the SIE protocols (*F*(2, 22) = 11.2, *p* = 0.001, $\eta_{{\text{p}}}^{2}$ = 0.51). Total work in the SIE10 protocol was 4.7% higher than in SIE30 (*p* = 0.006, ES = 1.15) and 2.6% higher than in SIE15 (*p* = 0.005, ES = 1.20). However, no difference in total work was observed between the SIE30 and SIE15 protocols (*p* = 0.235, ES = 0.55) (Table 4).

#### Minimum power

Although differences in minimum PO were observed among the SIE protocols (*F*(2, 22) = 4.94, *p* = 0.017, $\eta_{{\text{p}}}^{2}$ = 0.310), pairwise comparisons did not reveal any differences (*p* = 0.051–0.78) (Table 4). Only an unclear difference in minimum PO was observed between the SIE30 and SIE10 protocols (*p* = 0.051, ES = 0.81).

#### Fatigue Index

No differences in FI% were observed among the SIE protocols (*F*(2, 22) = 0.421, *p* = 0.662, $\eta_{{\text{p}}}^{2}$ = 0.037) (Table 4).

## Discussion

It is suggested that performing SIE sessions that elicit high PPO_SIE_ while spending as much time as possible at intensities close to ${\dot{\text{V}}}$O_2max_ is important for optimizing endurance performance (Shi et al. 2018). Nevertheless, the optimal work to recovery ratio to maximize these specific elements during SIE protocols has yet to be determined (La Monica et al. 2016; Lloria-Varella et al. 2023; Shi et al. 2018; Vardarli et al. 2021). The main finding of the present investigation was that both novel clustering methods (SIE15 and SIE10) were effective in achieving greater PPO_SIE_, ${\dot{\text{V}}}$O_2peak_, and percent of ${\dot{\text{V}}}$O_2max_ responses when compared to the traditional SIE30 protocol. This has valuable practical implications as, with endless potential combinations the “optimal” work to recovery ratio to maximize PPO_SIE_ and time spent at a high % of ${\dot{\text{V}}}$O_2max_, our data demonstrate that introducing short breaks that allow for partial recovery during each bout of a SIE session elicits simultaneously greater physiologic and mechanical responses overall.

One key factor that may differentiate the responses between the experimental protocols and the traditional SIE30 protocol could be the potentially enhanced reperfusion facilitated by the introduction of recovery periods within the sprint bouts. A sudden decrease in PPO_SIE_ and average PO values during an all-out effort in SIE30 protocol is known to be associated with occlusion and perfusion (Buchheit et al. 2012). In the SIE30 protocol, as repetitions progress under high load (7.5% of BM), there is a decrease in angular speeds and inertia, which negatively affects occlusion during the efforts and perfusion during recovery periods. This has a detrimental effect in hemodynamic responses contributing to oxidative phosphorylation processes, especially during recovery periods (Buchheit et al. 2012). Compared to the SIE30 protocol, the cluster recovery periods featured in the experimental protocols may enhance reperfusion, thereby facilitating the recovery of PCr, the clearance of metabolic by-products, and the restoration of oxygen levels, which can potentially have a favorable impact in physiologic and mechanical responses. Therefore, providing a short “intra” bout recovery time may not only result in reduced depletion of PCr stores but also allow for partial resynthesis of PCr, which, in turn, allows for greater PO generation in consequent sprint exercise within the clustered bouts. Given that ~ 50% of PCr stores are restored within the first ~ 20-s after maximal efforts (Bogdanis et al. 1995; Harris et al. 1976), the balance between PCr depletion and resynthesis remains favorable, even with shorter SIE bouts. Simultaneously, a reduced inorganic Phosphate (Pi) accumulation in SIE15 and SIE10 compared to SIE30 may contribute to lower muscle fatigue and enhanced contraction efficiency. Therefore, the combined effects of more rapid PCr replenishment and lower Pi accumulation in SIE15 and SIE10 may explain the superior performance outcomes observed in these protocols compared to SIE30.

Studies have suggested that the beneficial adaptations gained through SIE interventions were likely connected to producing high PPO_SIE_ values during the initial seconds of each work bout, rather than the total work done (Hazell et al. 2010; Ross et al. 2016; for a review, see Vollaard et al. 2017). If this is the case, then our results are promising, as the SIE10 and SIE15 protocols not only resulted in a greater total amount of work done, but also a greater PPO_SIE_ compared to the SIE30 protocol. (Table 4). These results suggest that emphasizing high PPO_SIE_ at the start of each work bout could be critical for optimizing performance outcomes in SIE protocols. The SIE15 and SIE10 protocols, which provided greater PPO_SIE_ with the clustering method, indicate that cluster recovery periods (10-s for SIE10 and 15-s for SIE15) between all-out efforts may offer an appropriate balance between power generation and fatigue management. Previous studies have also considered sprint exercise modalities that included short work durations with a variety of recovery interventions. For example, Danek et al. (2020) compared a SIE (6 repetitions × 10-s sprints with 4-min active recovery in between repetitions) to a repeated multi-set RSE (2 sets × 3 repetitions × 10-s sprints with 18-min active recovery between sets). Compared to SIE, the multi-set RSE protocol elicited larger minimum PO, greater total work, and lower percent FI values than SIE. However, similar PPO_SIE_ responses were obtained from both protocols. These results indicate that the differences in the FI from the higher minimum PO rather than the PPO_SIE_ responses. Although our findings showed that PPO_SIE_ responses were higher in the SIE15 and SIE10 protocols, the similar FI observed can be attributed to the fact that minimum PO values were similar but slightly higher. This is because, despite the similarity in minimum PO values, an increase in PPO_SIE_ leads to an increase in FI.

While these findings highlight the acute effects of different sprint protocols, it is also important to consider how these protocols may contribute to long-term adaptations. Ikutomo et al. (2018) compared two multi-set repeated sprint training (RST) protocols differentiated by shorter and longer recovery duration over a 3-week period, with training sessions performed three times per week. When comparing PPO_SIE_ across the training sessions, the long-recovery multi-set RST group exhibited greater PPO_SIE_ during sprints 4–12, likely due to the additional recovery time provided after every three sprints. Their results also demonstrated that the RST protocol with longer recovery periods resulted in a greater improvement in PPO_SIE_ compared to the shorter recovery multi-set RST protocol after training. On the other hand, Hazell et al. (2010) demonstrated that the 4 × 10-s and 4 × 30-s SIT protocols similarly increased ${\dot{\text{V}}}$O_2max_ with 4-min active recovery periods after 2 weeks of SIT (Hazell et al. 2010). Although 30-s bouts were more effective than 10-sbouts in enhancing aerobic metabolism, the study showed that SIT protocols involving both bout durations resulted in similar improvements in ${\dot{\text{V}}}$O_2max_. They speculated that the main reason for providing these similar gains was that the 10-s sprint protocol produced higher PPO_SIE_ values. Supporting this, in a training study where two groups performed 4–6 × 15-s long all-out repetitions with 4 min and 45-s active recovery periods, or 30-s all-out bouts with 4 min and 30-s active recovery periods for 4 weeks, Zelt et al. (2014) showed that both groups achieved increases in ${\dot{\text{V}}}$O_2max_, by 8% and 5%, respectively. Similarly, Lloria-Varella et al. (2023) showed that 8–12 repetitions of 15-s all-out efforts with 2 min active-to-passive transition recovery periods elicited an ~ 8% increase in ${\dot{\text{V}}}$O_2max_ as well as a greater total work (indicating a greater PO) after 2 weeks of SIT, with no further increases thereafter (i.e., after 4 weeks of training). Interestingly, the lack for further increases in ${\dot{\text{V}}}$O_2max_ in Lloria-Varella’s study (2023) was accompanied by the absence of increases in total work and, thus, PO during the all-out bouts, suggesting a connection between a plateau in total work and ${\dot{\text{V}}}$O_2max_. If sustaining greater POs is important for aerobic adaptations during SIE, then our study provides evidence to support the use of both experimental protocols, with higher PPO_SIE_, average PO, and greater total work compared to the SIE30 protocol.

In addition to a high PPO_SIE_, a high ${\dot{\text{V}}}$O_2_ response and time spent at a high %${\dot{\text{V}}}$O_2max_ are considered important acute responses to maximize aerobic adaptations (Shi et al. 2018). However, published research to-date has not been able to achieve a simultaneous combination of a high PPO_SIE_ response, a high ${\dot{\text{V}}}$O_2_ response, and a prolonged time spent at a high %${\dot{\text{V}}}$O_2max_. For example, La Monica et al. (2016) applied three different RSE protocols which were 10 × 6-s of maximal efforts with 1:2 (12-s active recovery), 1:3 (18-s active recovery) and 1:4 (24-s active recovery) work to recovery ratios. The VO_2_ response during recovery periods was 12% and 20% higher in the 1:2 SIE protocol compared to the 1:3 and 1:4 respectively (La Monica et al. 2016). As recovery time decreased, VO_2_ during recovery periods increased, while total work and average PO decreased. Further, Shi et al. (2018) investigated the effect of different passive recovery durations by applying 40 repetitions of 6-s maximal efforts with 1:2.5 (15-s recovery), 1:5 (30-s recovery), and 1:10 (60-s recovery) work to recovery ratios. Their results showed that when the recovery duration shortened, time spent ≥ 80% ${\dot{\text{V}}}$O_2max_ increased, but PPO_SIE_ and average PO also decreased to a greater extent. In the present study both experimental protocols resulted in a higher PPO_SIE_ response compared to the SIE30 protocol, as well as higher ${\dot{\text{V}}}$O_2peak_ responses and a longer time spent at ≥ 80% ${\dot{\text{V}}}$O_2max_ and ≥ ${\dot{\text{V}}}$O_2RCP_ when compared to the SIE30 protocol. The ${\dot{\text{V}}}$O_2peak_ response during SIE15 (96.1% ${\dot{\text{V}}}$O_2max_) and SIE10 (94.2% ${\dot{\text{V}}}$O_2max_) protocols were 11% and 8.3% greater than the response in the SIE30 protocol (86% ${\dot{\text{V}}}$O_2max_), respectively. In addition, the time spent at ≥ 80% of ${\dot{\text{V}}}$O_2max_ and ≥ ${\dot{\text{V}}}$O_2RCP_ in the SIE15 and SIE10 protocols was ~ 2.5 times longer than that of the SIE30 protocol (Table 1). ${\dot{\text{V}}}$O_2_ in the cluster recovery period of 10 or 15 s in work bout clusters of SIE10 and SIE15 protocols had less time to recover toward baseline values compared to SIE30 (Table 2). In fact, ${\dot{\text{V}}}$O_2_ work bout and recovery responses in the SIE15 and SIE10 protocols were similar, which explains the longer times spent at at ≥ 80% of ${\dot{\text{V}}}$O_2max_ and ≥ ${\dot{\text{V}}}$O_2RCP_ (Tables 1, 2, 3).

Our findings are consistent with previous studies on the development of ${\dot{\text{V}}}$O_2_ kinetics during and after maximal-intensity efforts. These indicate that ${\dot{\text{V}}}$O_2_ kinetics increase very rapidly throughout a 30-s all-out effort (Beneke et al. 2002; Ozkaya et al. 2024) and that peak ${\dot{\text{V}}}$O_2_ is attained early in the recovery period following completion of the effort (Ozkaya et al. 2024). In other words, a higher peak ${\dot{\text{V}}}$O_2_ value is achieved during the recovery period following a sprint effort within the extreme exercise domain. For example, Ozkaya et al. (2024) demonstrated that ${\dot{\text{V}}}$O_2_ kinetics increased rapidly during a 30-s sprint against 7.5% of BM and continued to rise during the first 10–20 s of the recovery period, ultimately reaching peak ${\dot{\text{V}}}$O_2_. These responses provide valuable insights for structuring SIE protocols and manipulating acute physiological responses. Indeed, these findings are also consistent with exercise sessions based on RSE and SIE. These responses provide valuable insights for the structuring of SIE protocols and the monitoring of acute physiologic responses. Hazell et al. (2014) observed higher ${\dot{\text{V}}}$O_2_ responses during the recovery periods of SIE protocols with similar work and recovery durations. They applied 30-s all-out work bouts against 10% BM for 4 repetitions with 4-min active recovery periods (Hazell et al. 2014). The ${\dot{\text{V}}}$O_2_ response in the first 30 s of the recovery period was greater than the ${\dot{\text{V}}}$O_2_ response during the work bout, corresponding to 88–99% ${\dot{\text{V}}}$O_2max_ (Hazell et al. 2014). Although the 30-s work period is dominant in terms of aerobic metabolism compared to 15 and 10-s work periods, the reason SIE15 and SIE10 protocols may result in more time spent at high % of ${\dot{\text{V}}}$O_2max_ and ${\dot{\text{V}}}$O_2peak_ compared to SIE30 could be due to more frequent cluster recovery periods. The results of Ulupınar and colleagues’ RSE study support the interpretation of our findings (Ulupınar et al. 2023). The study investigated the contributions of energy systems, lactate and ${\dot{\text{V}}}$O_2_ responses during passive recovery periods across four different RSE equalized sprint distance protocols. Protocols comprised combinations of 15 and 30 m sprints with 30-s passive recovery periods, and a 1:5 work to recovery ratio (15 m sprints with 12-s passive recovery and 30-m sprints with 22-s passive recovery, respectively). Results demonstrated higher ${\dot{\text{V}}}$O_2_ responses during the recovery periods in protocols with a 1:5 work to recovery ratio compared to those with 30-s passive recovery periods. Importantly, their findings revealed that ATP-PCr stores were replenished most rapidly (~ 15–20 s) in the early phase of the recovery period and that as the recovery duration increased, ${\dot{\text{V}}}$O_2_ decreased, thereby reducing the rate of PCr resynthesis. This suggests that in a protocol optimized with appropriate work to recovery ratios, shorter recovery periods may enhance the efficiency of the ATP-PCr system while also enhancing the aerobic contribution. Notably, despite the protocols with a 1:5 work to recovery ratio showing a faster PCr recovery and a higher aerobic contribution, they resulted in a similar or even greater lactate response.

Although the three SIE protocols in our investigation were comparable in total exercise and recovery duration, the clustered sprint bouts and cluster recovery periods in SIE10 and SIE15 could have resulted in higher absolute phosphagen, glycolytic, and aerobic energy demands compared with SIE30. Indeed, despite using different sprint durations (10 s, 15-s, and 30 s), all SIE protocols elicited similar [La_max_] and ΔLa responses. This indicates that shorter, but more sprint repetitions can also elicit similar glycolytic responses, and that the metabolic stress patterns on energy systems are shaped not only by total duration but also by sprint structure and recovery strategies. Consistent with this, previous studies have shown that variations in exercise duration, recovery time, and work to recovery ratios in SIE or RSE protocols can modify the interaction among different energy systems (Fiorenza et al. 2019; Ulupınar et al. 2023; Tortu et al. 2024). Consequently, although similar [La_max_] responses were observed across the three SIE protocols, the SIE10 and SIE15 protocols may lead to higher PPO_SIE_, greater ${\dot{\text{V}}}$O_2peak_, and a longer duration spent at higher %${\dot{\text{V}}}$O_2max_.

There are some experimental considerations to highlight. In this study, we used the time spent above the ${\dot{\text{V}}}$O_2_ value corresponding to the RCP, in addition to the time spent at ≥ 80% of ${\dot{\text{V}}}$O_2max_, as a reference threshold to define ‘high percentages’ of ${\dot{\text{V}}}$O_2max_. This threshold was established to facilitate the comparison of metabolic demands between different SIE protocols. Admittedly, this is a somewhat arbitrary value, especially considering that: (i) current data indicate that adaptations to endurance exercise training are based on the metabolic perturbations induced by the intervention, which is closely associated with the exercise intensity domain being elicited (Inglis et al. 2024); and (ii) the % of ${\dot{\text{V}}}$O_2max_ at which the second threshold occurs varies between participants (ranging from ~ 75 to 95% of ${\dot{\text{V}}}$O_2max_) (Iannetta et al. 2020). However, we accepted this approach to be in line with previously related literature (Burgomaster et al. 2005; Freese et al. 2013; Shi et al. 2018), while considering that the main point of metabolic disruption generated by SIE30 compared to SIE15 and SIE10 is lower remains valid. It is also important to note that the participants in this study were exclusively males. This was not a deliberate aspect of the study design but rather a consequence of the unanticipated lack of female volunteers during the recruitment process.

## Conclusion

Although the load, work bout and recovery durations in the three SIE protocols were equalized, the PO and ${\dot{\text{V}}}$O_2_ responses were greater during SIE15 and SIE10 compared to SIE30. By maximizing the acute responses associated with aerobic adaptations, the SIE15 and SIE10 protocols may offer a more advantageous approach for maximizing increases in ${\dot{\text{V}}}$O_2max_ compared to the traditionally applied SIE30 protocol. This study highlights the practical benefits of implementing a clustering method in SIE protocols for maximizing time spent at ${\dot{\text{V}}}$O_2max_ and increasing PPO_SIE_ during a session. Coaches and practitioners should consider incorporating the SIE10 or SIE15 protocol, which utilizes a clustering approach, into training regimens to optimize longer time spent at or near ${\dot{\text{V}}}$O_2max_, higher PPO_SIE_, and ${\dot{\text{V}}}$O_2_. For example, athletes preparing for short-duration, maximal-intensity sports such as sprinting or cycling sprints may benefit from the SIE10 protocol due to its emphasis on explosive power. Conversely, endurance athletes may find the SIE15 protocol more suitable as it provides a balance between power generation and aerobic strain. These protocols can also be particularly beneficial for athletes involved in sports requiring repeated maximal-intensity efforts, such as football, basketball, rugby, among others. By adjusting the work to recovery ratios and cluster durations, training programs can be tailored to meet specific performance goals, whether focusing on improving overall aerobic and anaerobic training adaptations.

## Data Availability

Data generated and/or analyzed during this study are available from the corresponding author upon reasonable request.

## Abbreviations

%${\dot{\text{V}}}$O_2__max_: Percentage of maximal oxygen uptake
[La_max_]: Maximum blood lactate
BM: Body mass
ES: Effect size
GXT: Maximal graded exercise test
HR_max_: Maximal heart rate
HR_peak_: Peak heart rate
PCr: Phosphocreatine
Pi: Inorganic phosphate
PO: Power output
PPO_SIE_: Peak power output obtained from sprint interval exercise
PPO_GXT_: Peak power output obtained from maximal graded exercise test
r: Recovery
RCP: Respiratory compensation point
RPE: Rating of perceived exertion
RPM: Revolutions per minute
RSE: Repeated sprint exercise
RST: Repeated sprint training
s: Second
SIT: Sprint interval training
SIE: Sprint interval exercise
SIE10: 4 × (3 × 10-s work bout), cluster recovery: 10-s and long recovery: 220-s
SIE15: 4 × (2 × 15-s work bout), cluster recovery: 15-s and long recovery: 225-s
SIE30: 4 × 30-s work bout, recovery: 240-s
$${\dot{\text{V}}}$$O_2_: Oxygen uptake
${\dot{\text{V}}}$O_2average_: Average oxygen uptake during work bout or recovery period
${\dot{\text{V}}}$O_2max_: Maximal oxygen uptake
${\dot{\text{V}}}$O_2peak_: Peak oxygen uptake obtained from sprint interval exercise
${\dot{\text{V}}}$O_2RCP_: The ${\dot{\text{V}}}$O_2_ level corresponding to the respiratory compensation point
W: Watt
ΔLa: Delta blood lactate
$\eta_{{\text{p}}}^{2}$: Partial eta squared
