# Correction: Physiologic and mechanical responses to clustered vs. traditional sprint interval exercise approaches

**DOI:** 10.1007/s00421-025-06018-3

**Published:** 2025-11-27

**Authors:** Refik Çabuk, Yıldırım Kayacan, Juan Manuel Murias, Bettina Karsten

**Affiliations:** 1 Department of Coaching Education, Yaşar Doğu Faculty of Sport Sciences, Ondokuz Mayıs University, Samsun, Türkiye; 2 College of Health and Life Sciences, Hamad Bin Khalifa University, Doha, Qatar; 3 Faculty of Health, Pedagogy & Social Science, CBS University of Applied Science, Cologne, Germany; 4 Institute for Lifecourse Development, School of Human Sciences, Centre for Exercise Activity and Rehabilitation, University of Greenwich, London, United Kingdom

**Correction: European Journal of Applied Physiology** 10.1007/s00421-025-05857-4

In the original version of this article, author noticed a minor typo, in the second-to-last paragraph of the introduction section.

The relevant sentence is:

“Thus, dividing a 30-s work bout into 2 or 3 work bout clusters to provide recovery durations within sprints that allow for partial recovery of PCr while still maintaining a high cardiovascular strain might allow for greater PO values and % of ${\dot{\text{V}}\text{O}}_{2\max }$ or $\ge {\dot{\text{V}}\text{O}}_{2\text{RCP} }$ responses to be observed.”

In this sentence, the word “prove” should be “provide”.

The horizontal line separating the header row between "SIE30" and "Cluster 1" is missing in Table 2.

Table 2, which previously appeared as

**Table:** **Table 2** Comparison of the ${\dot{\text{V}}}$O_2peak_ response (mL kg^−1^ min^−1^) obtained from work bout and active recovery periods of clusters in each set in the SIE30, SIE15 and SIE10 protocols

Variables		SIE30	SIE15		SIE10		
Set	Period	Cluster 1	Cluster 1	Cluster 2	Cluster 1	Cluster 2	Cluster 3
1	Work bout	39.6 ± 6.6	32.0 ± 6.3^b^	43.5 ± 5.4^b^	36.0 ± 6.8	42.5 ± 5.5^c^	37.6 ± 9.1
	Recovery	37.3 ± 5.4	43.2 ± 7.9^b^	39.7 ± 6.6^b^	36.2 ± 8.2	38.4 ± 7.4^c^	35.7 ± 7.2
2	Work bout	45.3 ± 9.6^a^	39.8 ± 5.2^b^	45.8 ± 6.7^b^	34.7 ± 7.4^c^	43.7 ± 7.2	42.4 ± 6.2
	Recovery	38.3 ± 8.2^a^	45.1 ± 4.6^b^	40.7 ± 6.5^b^	42.8 ± 8.9^c^	40.9 ± 6.2	40.7 ± 6.7
3	Work bout	44.3 ± 7.1^a^	39.8 ± 5.8^b^	46.4 ± 7.2^b^	36.3 ± 4.5	42.8 ± 7.6	39.0 ± 6.9
	Recovery	37.0 ± 6.9^a^	47.0 ± 6.4^b^	40.3 ± 6.9^b^	42.7 ± 9.9	42.4 ± 5.4	39.4 ± 7.4
4	Work bout	42.2 ± 8.2^a^	41.1 ± 6.7	44.4 ± 7.8^b^	38.3 ± 5.1	42.6 ± 7.6	37.6 ± 4.9
	Recovery	34.7 ± 6.5^a^	44.3 ± 8.2	39.7 ± 7.4^b^	38.6 ± 6.1	42.3 ± 8.1	36.6 ± 6.8

^a^The highest ${\dot{\text{V}}}$O_2_ responses obtained from the work bout and active recovery period of each set in the SIE30 protocol

^b^The highest ${\dot{\text{V}}}$O_2_ responses obtained from the work bout and active recovery period of each set in the SIE15 protocol

^c^The highest ${\dot{\text{V}}}$O_2_ responses obtained from the work bout and active recovery period of each set in the SIE10 protocol

But Table 2 should have appeared as shown below

**Table 2:** Comparison of the ${\dot{\text{V}}}$O_2peak_ response (mL kg^−1^ min^−1^) obtained from work bout and active recovery periods of clusters in each set in the SIE30, SIE15 and SIE10 protocols

Variables		SIE30	SIE15		SIE10		
Set	Period	Cluster 1	Cluster 1	Cluster 2	Cluster 1	Cluster 2	Cluster 3
1	Work bout	39.6 ± 6.6	32.0 ± 6.3^b^	43.5 ± 5.4^b^	36.0 ± 6.8	42.5 ± 5.5^c^	37.6 ± 9.1
	Recovery	37.3 ± 5.4	43.2 ± 7.9^b^	39.7 ± 6.6^b^	36.2 ± 8.2	38.4 ± 7.4^c^	35.7 ± 7.2
2	Work bout	45.3 ± 9.6^a^	39.8 ± 5.2^b^	45.8 ± 6.7^b^	34.7 ± 7.4^c^	43.7 ± 7.2	42.4 ± 6.2
	Recovery	38.3 ± 8.2^a^	45.1 ± 4.6^b^	40.7 ± 6.5^b^	42.8 ± 8.9^c^	40.9 ± 6.2	40.7 ± 6.7
3	Work bout	44.3 ± 7.1^a^	39.8 ± 5.8^b^	46.4 ± 7.2^b^	36.3 ± 4.5	42.8 ± 7.6	39.0 ± 6.9
	Recovery	37.0 ± 6.9^a^	47.0 ± 6.4^b^	40.3 ± 6.9^b^	42.7 ± 9.9	42.4 ± 5.4	39.4 ± 7.4
4	Work bout	42.2 ± 8.2^a^	41.1 ± 6.7	44.4 ± 7.8^b^	38.3 ± 5.1	42.6 ± 7.6	37.6 ± 4.9
	Recovery	34.7 ± 6.5^a^	44.3 ± 8.2	39.7 ± 7.4^b^	38.6 ± 6.1	42.3 ± 8.1	36.6 ± 6.8

^a^The highest ${\dot{\text{V}}}$O_2_ responses obtained from the work bout and active recovery period of each set in the SIE30 protocol

^b^The highest ${\dot{\text{V}}}$O_2_ responses obtained from the work bout and active recovery period of each set in the SIE15 protocol

^c^The highest ${\dot{\text{V}}}$O_2_ responses obtained from the work bout and active recovery period of each set in the SIE10 protocol

The original article has been corrected.

